# Primary pulmonary nuclear protein of the testis midline carcinoma: case report and systematic review with pooled analysis

**DOI:** 10.3389/fonc.2023.1308432

**Published:** 2024-01-09

**Authors:** Dong Zhao, Wei Cao, Shiqian Zha, Yixuan Wang, Zhou Pan, Jingyi Zhang, Ke Hu

**Affiliations:** ^1^Department of Respiratory and Critical Care Medicine, Renmin Hospital of Wuhan University, Wuhan, China; ^2^Office of Cancer Screening, National Cancer Center/National Clinical Research Center for Cancer/Cancer Hospital, Chinese Academy of Medical Sciences and Peking Union Medical College, Beijing, China

**Keywords:** pulmonary, NUT midline carcinoma (NMC), survival, pleural effusion, nuclear protein of the testis

## Abstract

Nuclear protein of the testis (NUT) midline carcinoma (NMC) is a rare tumor, with particularly low incidence in the lungs, and a correspondingly poor prognosis. To determine the clinicopathological characteristics, outcomes, and prognostic factors of primary pulmonary NMC, a case was reported and a systematic review was performed. Twenty-nine records, including ours, involving 62 cases, were finally included. The median age at diagnosis was 29.5 years. At presentation, the most common symptoms at presentation were cough (47.50%) and chest/back pain (37.50%). In terms of diagnosis, 32.14% of NMC cases were identified through immunohistochemistry (IHC); However, a greater number of cases may be misdiagnosed initially, and ultimately, the diagnosis of NMC was confirmed through a combination of IHC and fluorescence *in situ* hybridization (FISH). Despite the clinical application of various chemotherapy-based treatments, the actual effectiveness remains unsatisfactory. Furthermore, Cox regression analysis of multiple factors identified male gender and concurrent presence of pleural effusion as indicators of shorter survival time in patients. These results emphasize the importance of increased diagnostic awareness among clinical and pathology practitioners concerning NMC. While there is currently no established standard for treating NMC, a treatment approach combining multiple methods shows promise for future research. Concurrently, clinical and foundational investigations addressing variables such as gender and the presence of pleural effusion may yield valuable insights into the diagnosis and treatment of NMC.

## 1 Introduction

Nuclear protein of the testis (NUT) midline carcinoma (NMC) is a rare, extremely aggressive and poorly differentiated squamous cell carcinoma with a dismal prognosis (1, 2). In the most of patients, however, the disease is already advanced at the time of detection, leading rapid progression to death. While various treatments have been reported to be used after diagnosis, NMC remains a challenging clinical problem with limited treatment options (3–6).

Primary pulmonary NMC has been reported less frequently, and its prognosis is even more unfavorable (7). *Sholl et al.* reported the mOS was only 2.2 months (1). Many questions regarding the epidemiology, histopathology, clinical presentation, and treatments of primary pulmonary NMC remain unanswered. Herein, to help clinicians better recognize and understand this disease, we reported a cases from our group to describe the clinical characteristics of primary pulmonary NMC. An additional 61 cases from published studies were pooled to summarize the clinical characteristics, prognosis, and prognostic factors for the outcomes of patients with the primary pulmonary NMC.

## 2 Case report

**Figure 1:**
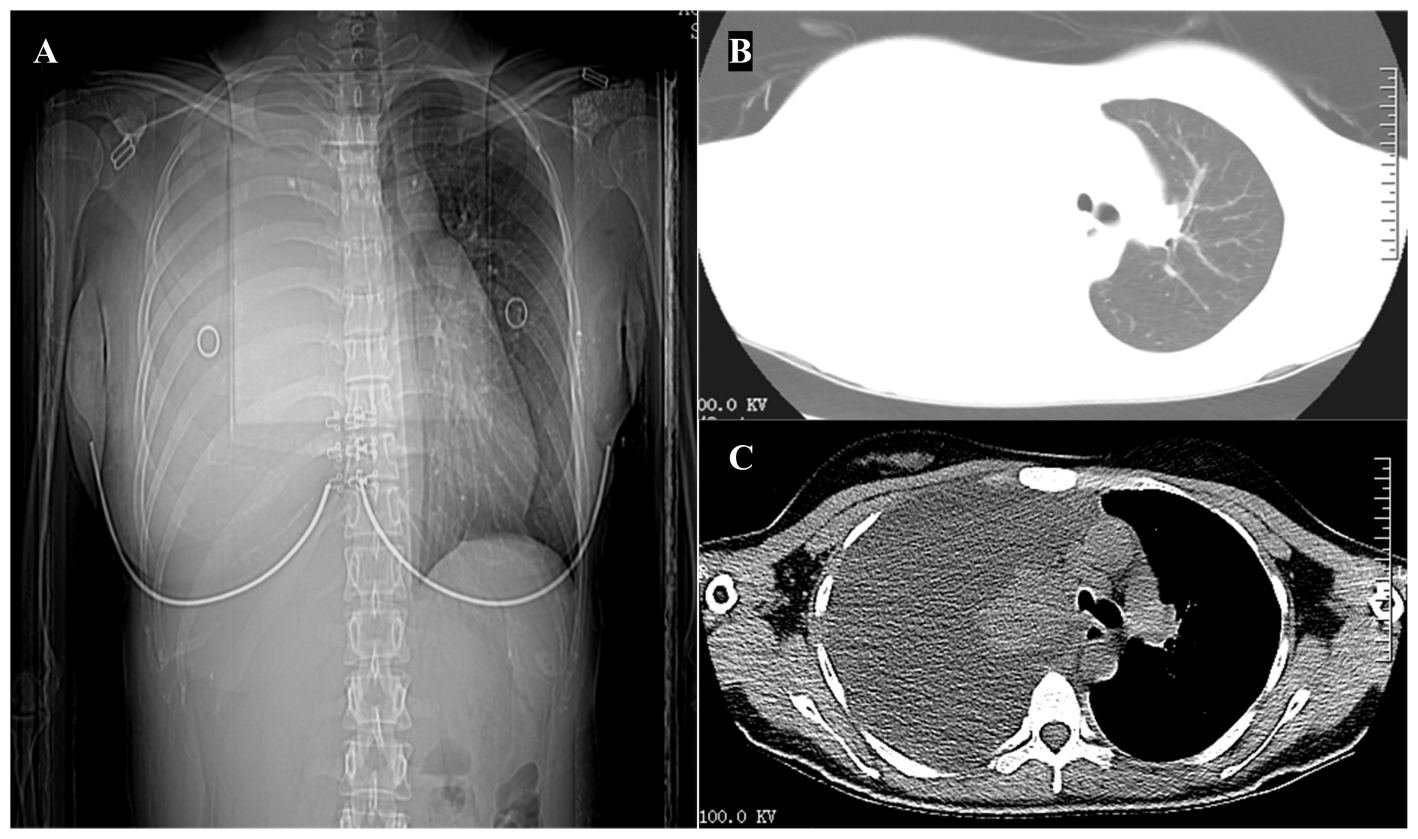
Imaging on hospitalization. **(A)** Chest x-ray demonstrated a right-sided pleural effusion. **(B, C)** Computed tomography scan of the chest showing a substantial amount of pleural effusion on the right side, with incomplete expansion of the right lung.

A 23-year-old female patient was admitted to the respiratory ward on June 8, 2020, with a complaint of “chest pain, cough for more than one month, aggravated with dyspnea for two weeks”. She had no significant medical history and denied a history of smoking. On admission, all other aspects of the physical examination, except for diminished breath sounds detected during auscultation of the right lung, revealed no abnormalities. A chest computed tomography (CT) scan showed a substantial amount of pleural effusion on the right side, with incomplete expansion of the right lung (**Figure 1**).

The patient underwent chest tube insertion for drainage. And the pleural fluid analysis revealed a red blood cell count of 0.009×10^12^/L, a total protein concentration of 37.1 g/L, an adenosine deaminase activity (ADA) of 7.87 U/L, a glucose concentration of 2.59 mmol/L, and a lactate dehydrogenase (LDH) activity of 390 U/L. The pleural fluid carcino-embryonic antigen (CEA) level was 204.68 ng/mL. The blood biochemistry results showed an albumin (ALB) level of 34.9 g/L, a globulin level of 15.7 g/L, a glucose level of 4.18 mmol/L, and a LDH level of 226 U/L. The serum CEA level was 16.72 ng/mL, the squamous cell carcinoma (SCC) antigen level was 1.32 ng/mL, and the neuron-specific enolase (NSE) level was 26.7 ng/mL.

**Figure 2:**
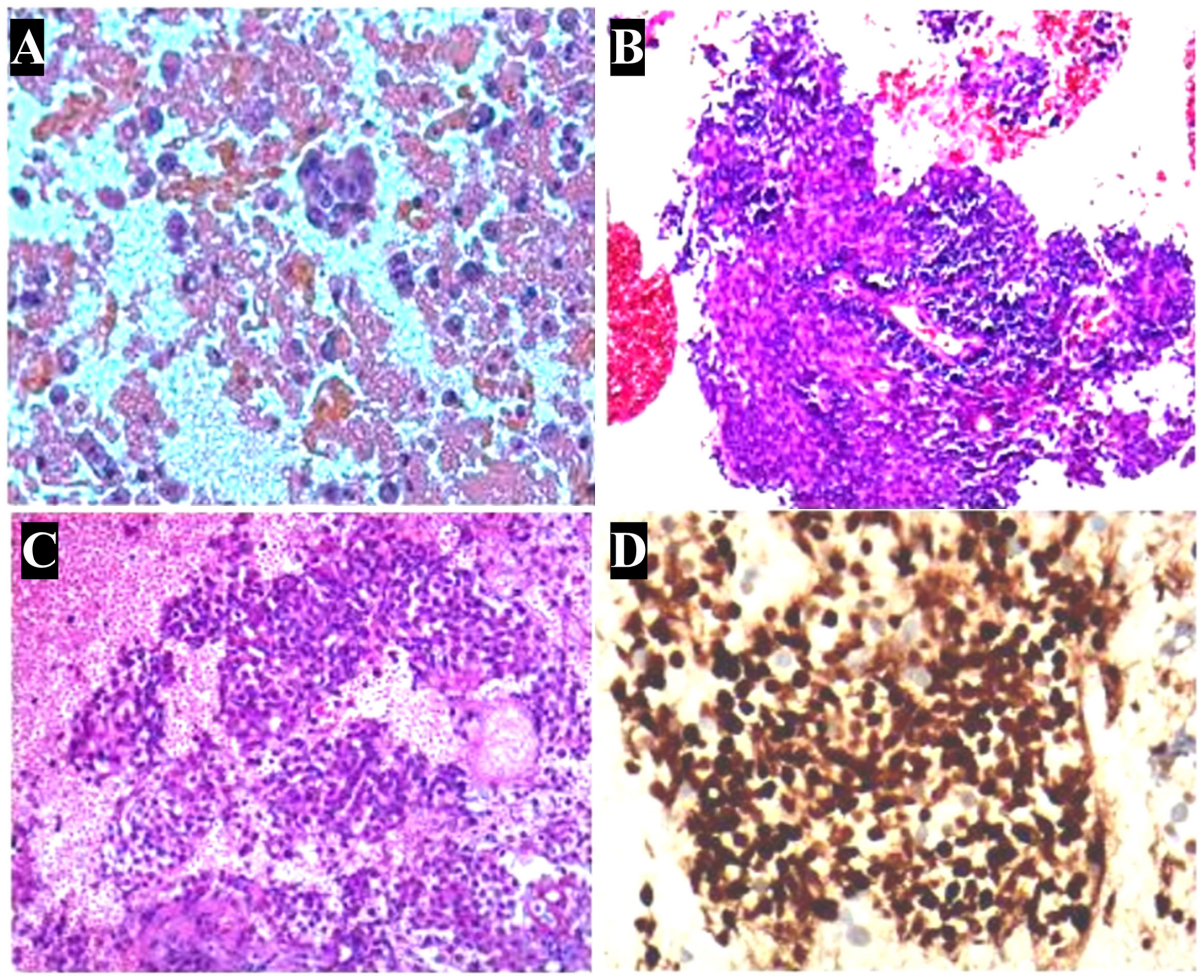
Pathologic characterization of tumor. **(A)** Hematoxylin and eosin stain of a cell block from pleural effusion sample shows a large number of cancer cells. **(B)** In aspiration cytology of a mediastinal lymph node, some tumor cells are medium-sized and poorly differentiated with scanty cytoplasm and hyperchromatic nuclei. **(C)** Biopsy of a mediastinal lymph node shows monotonous tumor cells and hyperchromatic nuclei. **(D)** Immunohistochemistry shows nuclear expression of NUT in the histology of a mediastinal lymph node. **(A-D)**: ×400.

Histopathological examination of the pleural effusion sample revealed the presence of cancer cells (**Figure 2A**). Immunohistochemical (IHC) analysis demonstrated positive expression of BerEP4, MOC31, p53, and CK7, CK5/6, EMA and P63, while negative expression of GATA-3, TTF-1, WT-1, ER, CDX-2, CK20, Napsin A, CD30, S-100, CD3, CD5, CD117, TdT and Villin. The preliminary pathological diagnosis, taking into account the results of the aforementioned immunohistochemistry, was a squamous cell carcinoma phenotype.

**Figure 3:**
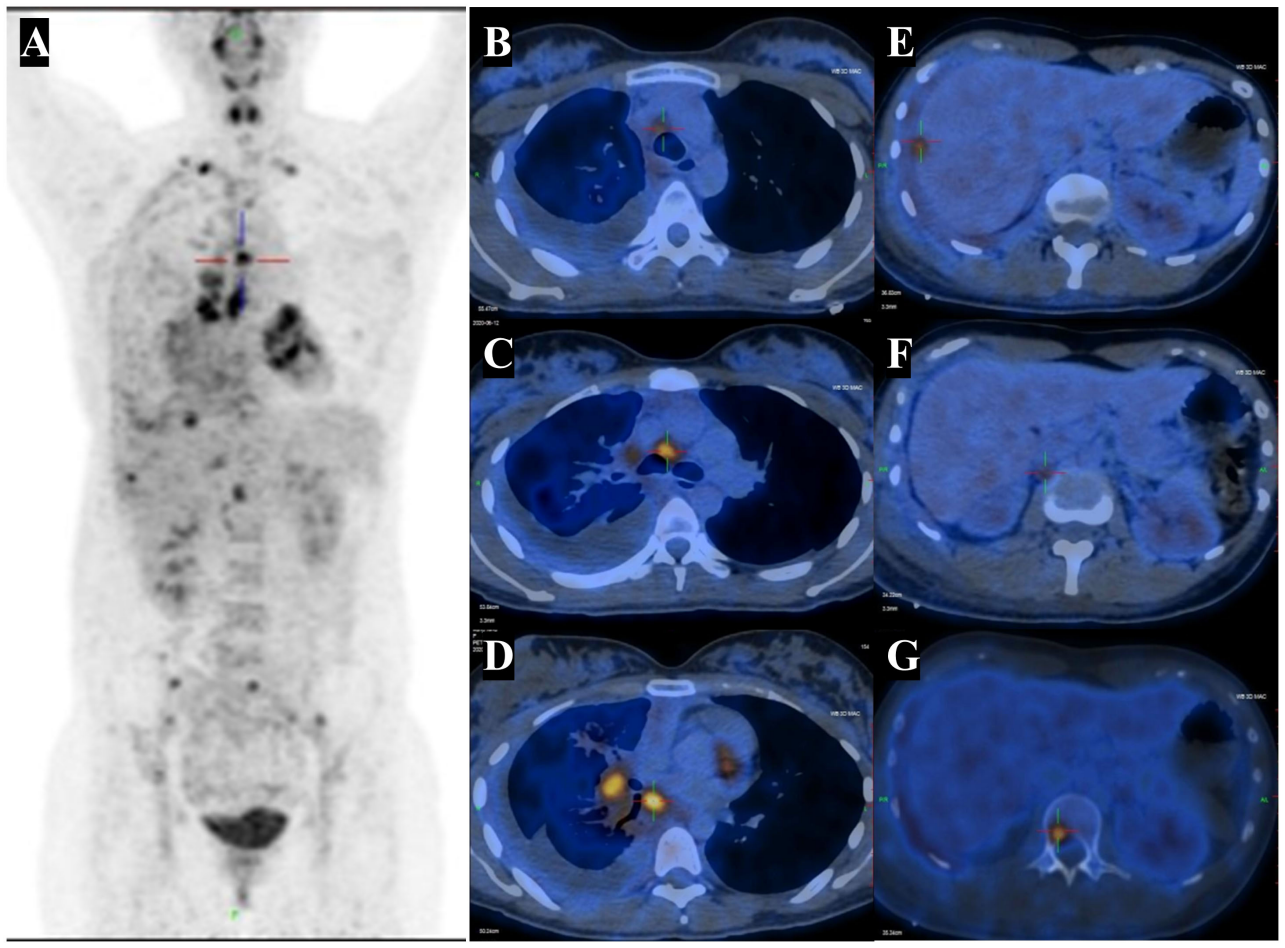
PET-CT highlights multiple hypermetabolic lesions. **(A)** Fused coronal PET-CT image shows an high metabolic foci in right lung cancer with lymph node, pleural and liver capsule, bone, as well as FDG-avid lesions in the right pleural effusion. **(B-D)** The right hilar and mediastinal lymph nodes with abnormal FDG uptake. SUVmax 4.4 - 7.8. **(E)** The abnormal FDG uptake in hepatic capsule with SUVmax 4.6 - 5.6. **(F)** The abnormal FDG uptake in retroperitoneal lymph nodes with SUVmax 3.4 - 3.6. **(G)** The abnormal FDG uptake in bone with SUVmax 3.9 - 5.8. CT, computed tomography; PET, positron emission tomography; FDG, fluorodeoxyglucose. SUVmax, maximum standardized uptake value.

Given the suspicion of malignant pleural effusion, an electronic bronchoscopy and ultrasound bronchoscopy-guided transbronchial needle aspiration (EBUS-TBNA) procedure was performed on June 15, 2020. The results revealed the presence of metastatic malignant tumors (**Figure 2B, C**) with a positive staining for NUT in the IHC analysis (**Figure 2D**), while CD117, CD5, TdT were negative, and Ki67 was positive with a rate of 30%. Based on clinical and IHC findings, the pathologist considered the possibility of NUT carcinoma (NMC) in the histological tissue. Additional molecular testing with NUT FISH confirmed the diagnosis of NMC ultimately. On June 12th, 2020, a positron emission tomography (PET)-CT scan was conducted to evaluate the patient’s disease staging (**Figure 3**). The results revealed multiple high metabolic foci with localized lesions in the bones, indicating malignant lesions.

**Figure 4:**
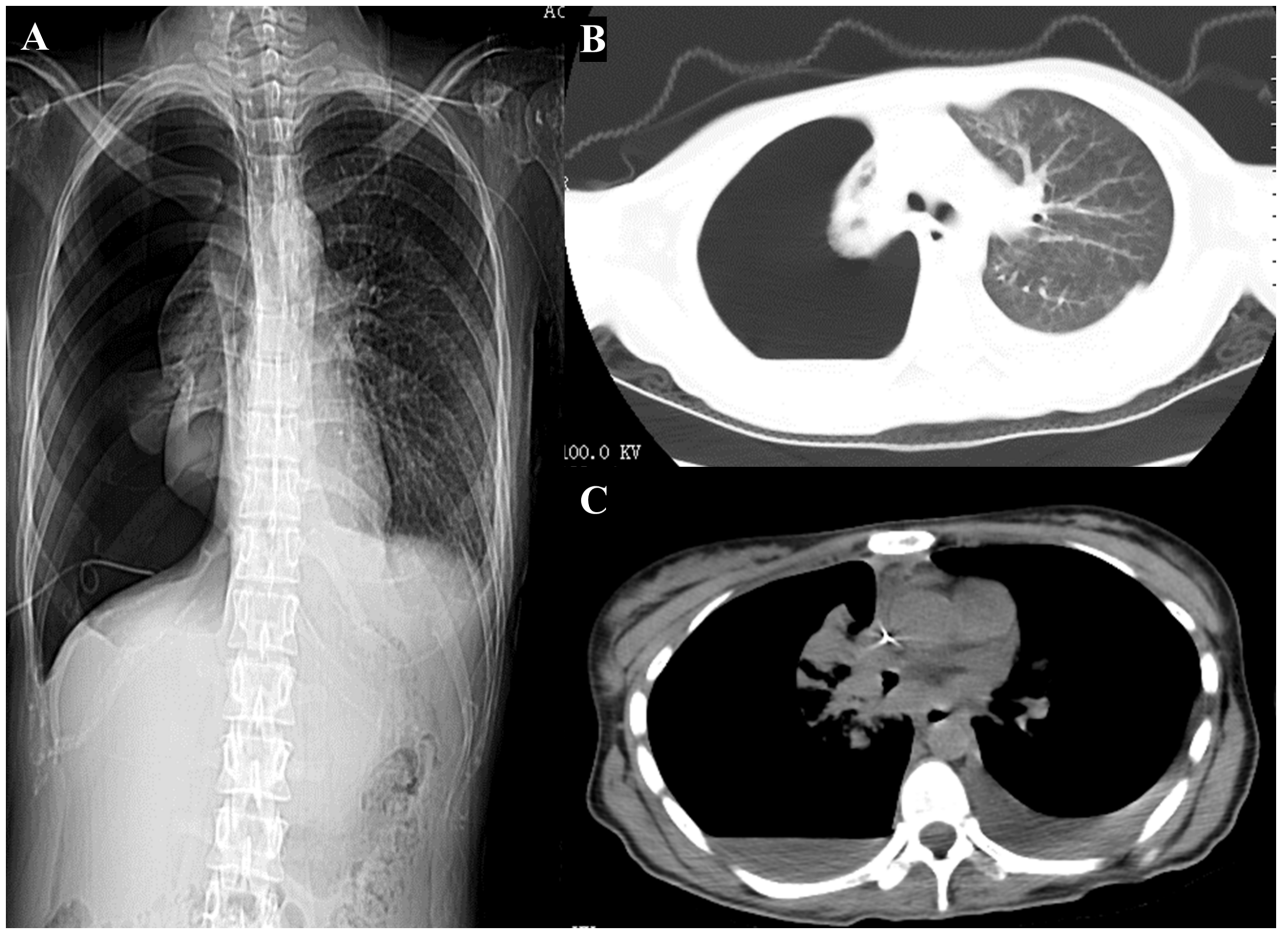
Computed tomography scan of the chest was performed during hers last hospitalization. **(A)** Chest x-ray imaging. **(B, C)** Computed tomography scan of the chest showing a right pneumothorax and left pleural effusion.

Due to the diagnosis of NMC stage IV, the patient underwent two cycle of chemotherapy (paclitaxel albumin-bound and carboplatin) combined with immunotherapy (cetuximab) on June 20, 2020 and July 11, 2020. On August 27, 2020 (the third hospitalization), the patient was admitted for the third cycle of treatment, with a CEA level of 58.71 ng/mL upon admission, which was higher than before. Due to the patient’s upper abdominal and lumbar pain, palliative radiotherapy was administered to the cone metastasis site (GTV1 at L3, L4, and S1 with 40Gy/4Gy/10f; GTV2 at T11 and L1 with 32Gy/4Gy/8f) on the basis of aforementioned treatment schemes. On September 2, 2020, the patient was readmitted for treatment evaluation and had an elevated CEA level of 75.34 ng/mL. The patient experienced continuous disease progression, as evidenced by a right pneumothorax and left pleural effusion on chest CT on December 10, 2020 (**Figure 4**). The patient died of respiratory failure on January 9, 2021, after an overall disease course of approximately 7 months.

The study protocol was approved by the ethics committee of Renmin Hospital of Wuhan University (Ethical Application Ref:2023K-K173). A written informed consent was obtained from the patient for the publication of any potentially identifiable images or data included in this article.

## 3 Systematic review

### 3.1 Methods

#### 3.1.1 Search strategy

At March 20, 2023, we conducted a literature search of the MEDLINE (PubMed) and Web of Science databases. The search terms included “nuclear protein of the testis”, “NUT” and “midline carcinoma” index words. Titles and abstracts were independently assessed by two reviewers (Wei Cao and Shiqian Zha). Any differences could be resolved through discussion or by hiring a third reviewer (Yixuan Wang) to reach consensus.

Inclusion criteria: (1) The literature should provide at least one of the survival data, other associated clinical characteristics or treatment. (2) Full text is available. (3) cases reported in the English literature. Exclusion criteria: (1) Literatures with repeated publications. (2) Reviews, meta-analysis, conference abstracts, or comment papers.

#### 3.1.2 Data extraction

Three researchers (Wei Cao, Shiqian Zha and Yixuan Wang) extracted the data independently, and any disagreements were identified and resolved through discussions with another author (Zhou Pan). We extracted the following data from the included studies: study characteristics (first author, year of publication, study period, number of patients), patient age, gender, other metastasis site, clinical presentation, primary cancer location, presenting symptoms, histopathologic features, survival time and so on.

#### 3.1.3 Statistical analysis

Analyses were conducted considering all included patients as one sample. Descriptive data were reported as median with interquartile range (IQR) or frequencies (%), respectively. Survival analysis was performed using Kaplan-Meier method. Univariate analysis was performed using Cox proportional hazards regression model, followed by a multivariate Cox regression analysis including only variables with a P value < 0.05 during univariate analysis. The analysis was performed using IBM SPSS statistical software package software (version 22.0, IBM Corp.) with a significance set at p < 0.05.

## 4 Results

### 4.1 Eligible studies

**Figure 5:**
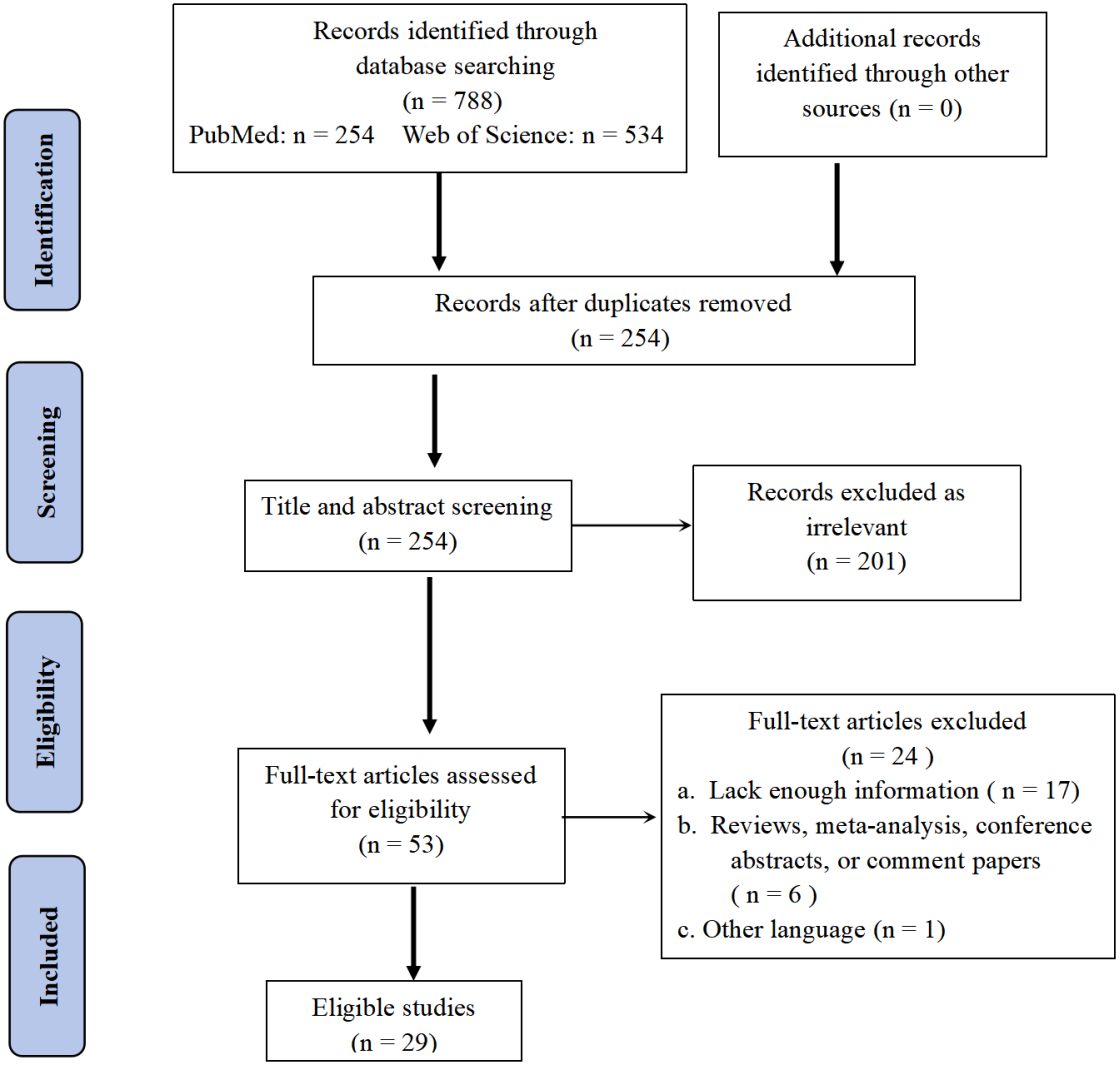
PRISMA (Preferred Reporting Items for Systematic Reviews and Meta-analysis) flow diagram of search strategy and study selection.

We conducted a comprehensive search of the literature and identified 254 articles for initial screening based on their titles and abstracts. Among these, 53 studies were selected for full-text review. After this process, we ultimately included 29 studies in our analysis, providing data on 61 cases of primary pulmonary NMC (**Figure 5**) (8–36). All of the studies included in our analysis provided information on survival times.

### 4.2 Characteristics of individual patient data

The main characteristics of 62 patients with Pulmonary NMC were summarized in the table below (**Table 1**). The median age at diagnosis was 29.5 years [Interquartile range (IQR): 18.5 - 36.8], with a female proportion of 46.77% (29/62). 24.19% of patients were diagnosed at less than 18 years old, while 70.97% were diagnosed between 18 - 60 years old. Among the patients with reported smoking status (n = 24), 54.17% were smokers. The primary locations of the tumor were distributed as follows: right upper lobe (16.36%), right lower lobe (16.36%), right middle lobe (3.64%), left upper lobe (7.27%), left lower lobe (14.55%), and hilar region (20.0%). The tumor size was < 2 cm in 0%, 2 - 5 cm in 20.0%, 5 - 10 cm in 45.45%, and > 10 cm in 18.18% of the patients. The most common symptoms at presentation were cough (47.50%) and chest/back pain (37.50%). Metastasis was detected in 42 reported patients, most commonly in the bone (73.81%). The 29 patients presented with pleural effusion in 45 reported cases (64.44%).

**Table 1:** Main characteristics of 62 patients of pumonary NMC.

Characteristics	Number of patients with reported results	Results
**Age at diagnosis, year, median (IQR)**	62	29.5 (18.5 - 36.8)
<18 year, %	62	15 (24.19%)
18-60 year, %	62	44 (70.97%)
>60 year, %	62	3 (4.84%)
**Females**, %	62	29 (46.77%)
**Smoker**, %	24	13 (54.17%)
Primary lung location^a^		
RUL, %	55	9 (16.36%)
RLL, %	55	9 (16.36%)
RML, %	55	2 (3.64%)
LUL, %	55	4 (7.27%)
LLL, %	55	8 (14.55%)
Hilum, %	55	11 (20.0%)
Tumor size (largest diameter)^b^		
<2 cm, %	55	0 (0%)
2-5 cm, %	55	11 (20.0%)
5-10 cm, %	55	25 (45.45%)
≥10 cm, %	55	10 (18.18%)
Presenting symptoms		
Cough, %	40	19 (47.50%)
Chest/back pain, %	40	15 (37.50%)
Dyspnea, %	40	8 (20.0%)
Shortness of breath, %	40	6 (15.0%)
Weight loss, %	40	5 (12.50%)
No symptom, %	40	1 (2.50%)
Distant metastasis		
Bone	42	31 (73.81%)
Bone marrow	42	3 (7.14%)
Liver	42	8 (19.05%)
Adrenal gland	42	6 (14.29%)
Brain	42	3 (7.14%)
Muscle	42	1 (2.38%)
Breast	42	2 (4.76%)
Ovary	42	2 (4.76%)
Heart (pericardium)	42	1 (2.38%)
No metastasis	42	3 (7.14%)
**Pleural effusion**, %	45	29 (64.44%)
Immunohistochemistry features		
CKpan (Pancytokeratin), %	13	12 (92.31%)
P63, %	23	22 (95.65%)
TTF-1, %	31	6 (19.35%)
CK6/7, %	12	8 (66.67%)
EMA, %	13	10 (76.92%)
CD99, %	17	9 (52.94%)
CK5/P40, %	9	8 (88.89%)
CD56, %	10	3 (30.0%)
Syn, %	9	4 (44.44%)
CD30, %	7	1 (14.29%)
Vimentin, %	6	4 (66.67%)
CAM5.2, %	4	2 (50.0%)
AE1/AE3 cytokeratins, %	21	16 (76.19%)
Ki-67 index, %, median (IQR)	11	60 (40-70)
Original Diagnosis		
NUT midline carcinoma, %	28	9 (32.14%)
Squamous cell carcinoma, %	28	4 (14.29%)
Primary neuroectodermal tumor, %	28	2 (7.14%)
Ewing’s sarcoma, %	28	1 (3.57%)
Undifferentiated carcinoma, %	28	5 (17.86%)
Epithelial carcinoma, %	28	2 (7.14%)
Small-cell lung carcinomas, %	28	3 (10.72%)
Non-small-cell lung carcinomas, %	28	2 (7.14%)
Treatment methods involved		
Surgery	60	5 (8.33%)
Chemotherapy	60	51 (85.0%)
Radiotherapy	60	26 (35.0%)
Target therapy	60	13 (21.67%)
Immunotherapy	60	2 (3.33%)
**Survival (month)**	62	4.85 (3.0-10.0)

^a^Out of the 55 patients with pulmonary masses described, 12 cases did not specify the exact location in the literature reports, but only mentioned whether the mass was located in the left or right lung. ^b^Out of 55 patients with pulmonary masses described, 9 cases did not have the size of the masses described in the literature report.

RUL, Right upper lobe; RML, Right middle lobe; RLL, Right lower lobe; LUL, Left upper lobe; LLL, Left lower lobe.

### 4.3 Histologic and immunohistochemical features

Several markers, including CKpan, P63, TTF-1, CK6/7, EMA, CD99, CK5/P40, CD56, Syn, CD30, Vimentin, CAM5.2, and AE1/AE3 cytokeratins were tested. The Ki-67 index, which indicates the level of cell proliferation, was also measured. The majority of patients (92.31%) had positive CKpan expression. And P63 was expressed in 95.65% of patients. The median Ki-67 index was 60%, with an IQR of 40% - 70%. NUT midline carcinoma was the most commonly diagnosed pathological type among the 28 reported cases, with a frequency of 32.14%. Nonetheless, several other pathological types were frequently misdiagnosed, including undifferentiated carcinoma (17.86%), squamous cell carcinoma (14.29%), small-cell carcinomas (10.72%), primary neuroectodermal tumor (7.14%), epithelial carcinoma (7.14%), non-small-cell carcinomas (7.14%), and Ewing’s sarcoma (3.57%).

### 4.4 Treatment strategies and clinical efficacy

**Figure 6:**
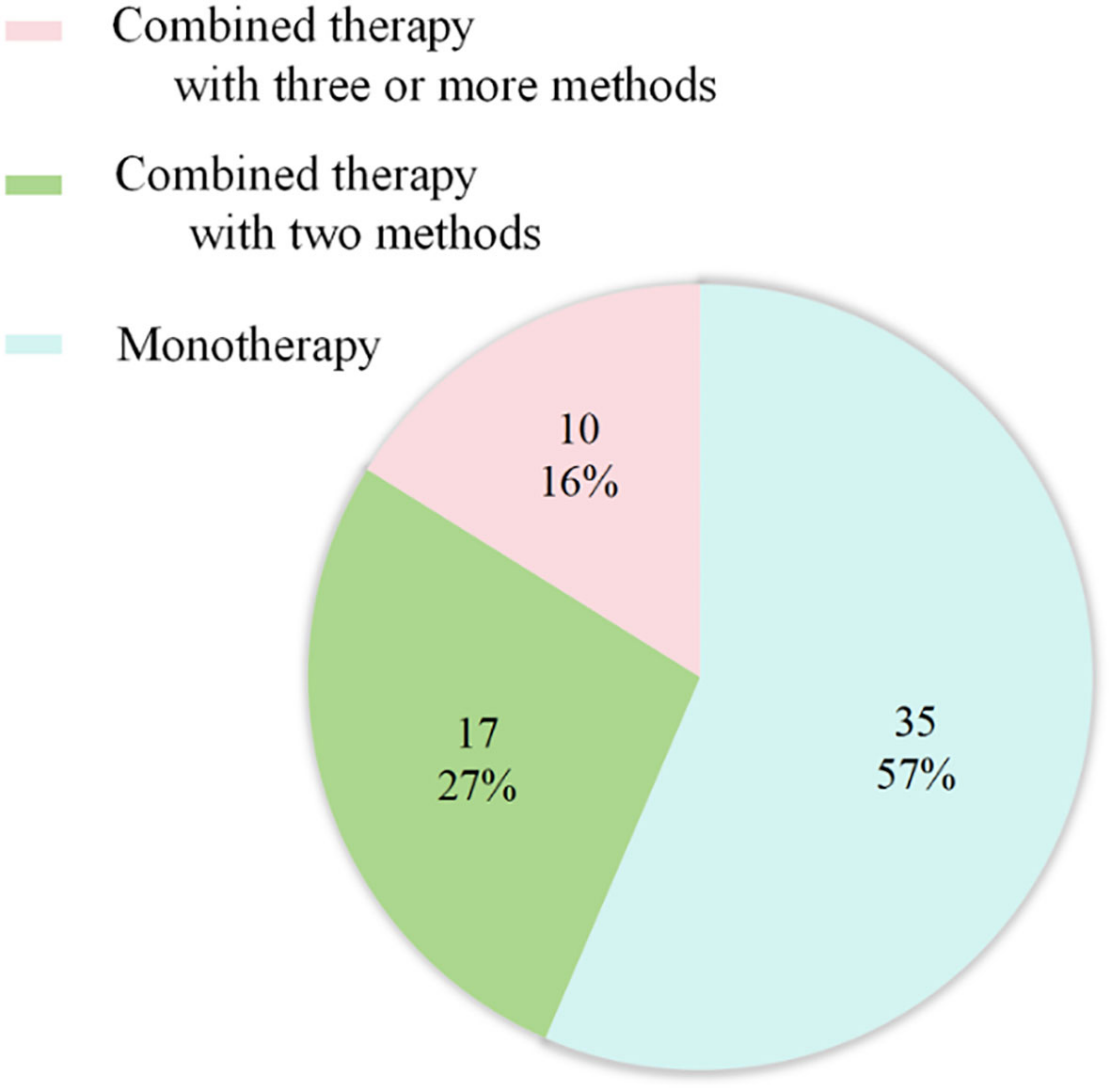
Treatment strategies for primary pulmonary NMC (n = 62). 35 cases received Monotherapy, of which 27 with chemotherapy; 17 cases treated with combined therapy with two methods, of which 11 with chemoradiotherapy. 10 cases treated with combined therapy with three or more methods, including 7 patients undergoing the combination of chemoradiotherapy with target therapy.

Regarding therapy, the majority of patients received chemotherapy alone (45%), chemoradiotherapy (CRT) (18.33%) and Combined targeted therapy with chemoradiotherapy (CRT) (11.67%) (**Figure 6**). The mOS was 4.85 months (IQR: 3.0 - 10.0 months) (**Table 1**).

### 4.5 Prognostic indicators for overall survival of patients with pulmonary NMC

As for survival after diagnosis of pulmonary NMC, univariate Cox analysis revealed that cases with males exhibited poorer prognosis than females [unadjusted hazard ratio (HR) 1.97, 95% CI: 1.16-3.33, P = 0.012], while cases with pleural effusion (unadjusted HR 2.68, 95% CI: 1.36–5.31, P = 0.005) or tumor size more than 10 cm (unadjusted HR 3.31, 95% CI: 1.30 - 8.44, P = 0.012) demonstrated worse outcomes than cases without pleural effusion or tumor size between 2 cm to 5 cm. However, age, smoking history, various biomarkers, site of metastasis, and different treatment methods were not significantly associated with survival (**Table 2**).

**Table 2:** Univariable and multivariable Cox models of characteristics that may affect survival to investigate risk factors for pulmonary NMC in 62 patients.

	Univariable models		Multivariable models	
	HR (95% CI)	p value	HR (95% CI)	p value
Age	0.99 (0.98 - 1.01)	0.183		
Male (vs. Female)	1.97 (1.16 - 3.33)	0.012	2.74 (1.10-6.87)	0.031
Smoker (vs. No Smoker)	0.76 (0.33 - 1.75)	0.519		
Pleural effusion (vs. None)	2.68 (1.36 - 5.31)	0.005	3.70 (1.52-9.03)	0.004
Tumor size (vs. 2 -5 cm) ^a^				
5 - 10 cm	1.28 (0.61 - 2.67)	0.518	0.53 (0.16-1.74)	0.294
> 10 cm	3.31 (1.30 - 8.44)	0.012	1.82 (0.49-6.75)	0.372
CK pan (vs. None)	0.74 (0.09 - 5.95)	0.774		
P63 (vs. None)	0.42 (0.05 - 3.22)	0.402		
TTF-1 (vs. None)	1.13 (0.45 - 2.81)	0.793		
CK6/7 (vs. None)	0.42 (0.09 - 2.10)	0.293		
CK5/P40 (vs. None)	3.47 (0.31 - 38.39)	0.311		
CD56 (vs. None)	1.84 (0.37 - 9.09)	0.454		
Syn (vs. None)	1.78 (0.34 - 9.25)	0.491		
EMA (vs. None)	0.70 (0.18 - 2.67)	0.596		
CD99 (vs. None)	2.49 (0.81 - 7.72)	0.113		
Vimetin (vs. None)	0.43 (0.05 - 3.93)	0.453		
AE1/AE3 (vs. None)	0.88 (0.32 - 2.47)	0.813		
Bone metastasis (vs. None) **^b^**	0.76 (0.36 - 1.58)	0.459		
Liver metastasis (vs. None) **^b^**	0.98 (0.45 - 2.16)	0.961		
Adrenal gland metastasis (vs. None) **^b^**	0.46 (0.18 - 1.16)	0.099		
Brain metastasis (vs. None) **^b^**	1.16 (0.35 - 3.79)	0.812		
Treatment methods (vs. Chemotherapy) ^c^				
CRT	0.53 (0.26 - 1.10)	0.089		
CRT+TT	1.40 (0.60 - 3.29)	0.438		

^a^No case that the tumor size < 2 cm was reported in there of the 62 patients. ^b^Despite pulmonary NMC patients frequently developed metastases at multiple sites, the most common metastatic sites of bone, liver, adrenal gland, brain were included into this study for Cox analysis. ^c^Althought different treatment modalities were reported, three predominant therapies were compared in this Cox analysis.

CRT, chemoradiotherapy; CRT+TT, a targeted therapy combined with chemoradiotherapy; HR, hazard ratio; CI, confidence interval.

**Figure 7:**
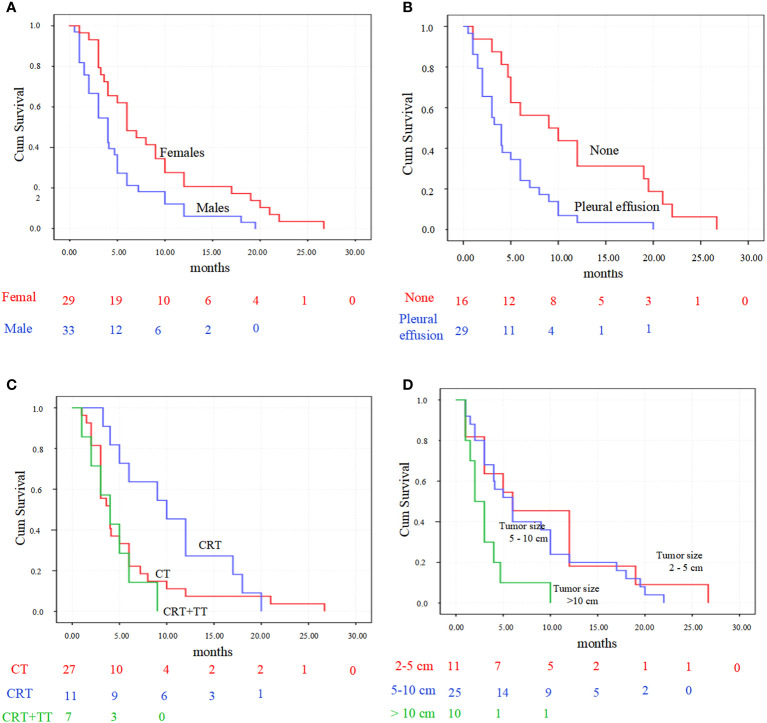
Kaplan-Meier survival analysis for primary pulmonary NMC patients. **(A)** patients’ overall survival accordingly with gender (*P* = 0.007). **(B)** survival was significantly longer in patient with pleural effusion than those without (*P* = 0.002). **(C)** overall survival comparison between the three predominant treatment strategies (*P* = 0.078). **(D)** overall survival comparison between the three tumor sizes (*P* = 0.011). CT, chemotherapy; CRT, chemoradiotherapy; CRT+TT, the combination of chemoradiotherapy with target therapy.

Kaplan-Meier analysis showed that gender (P = 0.007), tumor size (P = 0.011) and the presence of pleural effusion (P = 0.002) predicted a poor OS. While the different treatment methods (P = 0.078) exhibited not significantly associated with OS (**Figure 7**).

In multivariate Cox analysis, gender (HR 2.74, 95% CI 1.10 - 6.87, p = 0.031) and the presence of pleural effusion (HR 3.70, 95% CI 1.52 - 9.03, p = 0.004) remained significant predictors of survival after adjusting for other variables. Other factors were not significant.

## 5 Discussion

Primary pulmonary NMC is an exceedingly rare disease, and its pathogenesis remains unclear. Current evidence suggests that rearrangement of the NUT carcinoma family member 1 (NUTM1) gene located on chromosome 15 q l4 may be the predominant causative factor in the development of NMC (37). Consequently, positive results for NUT IHC or molecular testing techniques (such as FISH, PCR, and second-generation sequencing) are considered diagnostic criteria for NMC (30, 35, 38, 39). In this study, we conducted a comprehensive collection and analysis of cases of pulmonary NMC, with the objective of enhancing our understanding of the epidemiological, pathological, and immunohistochemical features of this disease.

In terms of epidemiology, primary pulmonary NMC mainly affected children and young adults, with a median age of 29.5 years (range: 6-74 years), younger than in other reports (38, 40). Both genders are equally affected. The tumors could be located in any segment of the lung, with about 50% of nodules measuring between 5-10 cm during diagnosis, indicating the onset of the disease was occult. And symptoms of pulmonary NMC were often non-specific, with cough being the most common symptom. Other symptoms might include chest pain, dyspnea, chest or back pain and so on.

Given the non-specific histological characteristics of NMC (2, 8, 28, 40, 41), most cases expressed p63, p40, CK6/7, EMA, CD99, Vimentin, and AE1/AE3 cytokeratins. In addition to these markers, NMC might also express TTF-1, CD56, Syn, CD30, and CAM5.2, which often leaded to misdiagnosis. For squamous cell carcinoma of the lung, which is typically associated with older male patients and a history of smoking. Conversely, NMC should be considered in the case of a non-smoking or younger patient (38). Becase midline structures was the common site for the occurrence of NMC, all thymic carcinomas should be evaluated for the possibility of NMC, and IHC could be utilized to identify NUT-positive cases. In this research, we reported a 23-year-old female with pleural effusion, which is positive for the squamous differentiation marker P63. However, given the absence of non-keratinizing thymic carcinoma metastasis, the possibility of NMC was considered. When tumor cells in NMC express Syn, misdiagnosis as small cell lung carcinoma might occur (42). However, small cell carcinoma is common in elderly patients with elevated serum NSE levels and is positive for CD56 and TTF-1, but negative for NUT, P63, P40 (43). But additionally, there are also some specific situations that need to be pointed out. The spectrum of small round blue cell tumours (including leukaemias) can enter the differential diagnosis in a subset of NMC expressing CD34 and CD99 and/or lacking expression of p40 and keratin (2, 44–46). NMC may express CD99, leading to potential confusion with extraskeletal Ewing’s sarcoma (46, 47). However, this malignancy is extremely rare in the lungs and mediastinum, warranting further molecular testing to confirm diagnosis. FISH testing can distinguish NMC from extraskeletal myxoid chondrosarcoma by the absence of EWSR1 breakage in NUTM1 (28, 48–50). The coexpression of TTF-1 with focal or multifocal p63 is not uncommon in certain lung tumors that simultaneously express neuroendocrine markers (1, 51), warranting consideration for the possibility of NMC. NMC also needed consideration in the differential diagnosis of poorly differentiated squamous cell carcinoma, small cell carcinoma, and combined small cell and squamous cell carcinoma, particularly when there is abrupt transition from small cell tumour areas to foci of squamous differentiation (11, 52). Therefore, when encountering the aforementioned specific situations again, the possibility of NMC should be considered. Concurrently, employ IHC for NUT protein immunohistochemical staining, and, contingent upon the specific context, undertake FISH molecular testing to facilitate discrimination (17, 38, 52).

NMC has yet to establish a standardized treatment protocol. Surgical resection, chemotherapy, and radiation therapy are proposed as potential treatments for primary pulmonary NMC (10, 16). Surgical intervention may be the preferred method for solid tumors and could significantly improve survival outcomes for patients in the early stage (10, 29). However, in this study, distant metastases were present in over 73.81% of patients, and only three cases were identified without any metastases. Thus, most NMC patients are diagnosed after the optimal surgical window has passed, making chemotherapy the primary treatment option. Nonetheless, there was controversy regarding the efficacy of various treatment modalities in prolonging survival and required larger sample size data for practical efficacy (10). Presently, various treatments based on tumor characteristics have been reported, including immunotherapy, targeted therapy, and anti-angiogenic therapy, as well as the combination of chemotherapy, immunotherapy (44, 53), targeted therapy (18, 54), and anti-angiogenic therapy (2). Despite this, the therapeutic effectiveness of these approaches has mainly been based on case reports or small sample studies. Targeted therapies, exemplified by bromodomain and extra-terminal Motif (BET) inhibitors, have attracted considerable attention and are perceived to hold promising prospects for clinical treatment (17). As BET proteins are crucial for the functioning of every cell in the body, and BET inhibitors are generally unable to selectively target cell types, targeting not only BRD4 but also BRD2 or BRD3, becomes imperative to minimize potential side effects (55, 56). Achieving selective targeting of cells by BET inhibitors would significantly alleviate these side effects.

In terms of survival, primary pulmonary NMC had a shorter survival time than overall NMC survival. Further analysis of factors affecting patient survival revealed that male gender and the presence of pleural effusion were high-risk factors for shorter OS. Although there was no significant difference in gender incidence, the reason for shorter survival in males needs further investigation. At present, there exists a paucity of research elucidating the specific mechanisms contributing to the diminished survival prognosis of male patients afflicted with NMC (9, 52, 56, 57). Delving into the following aspects could provide insights into the diminished survival rates observed in male NMC patients: disparate responses post-treatment, variations in hormonal levels, immune system reactions, and tumor staging at the time of diagnosis. Over half of the patients experienced pleural effusion in this study. The presence of pleural effusion generally indicated that tumor metastasis to the pleura and suggested disease progression to stage IV, with a worse prognosis. However, considering that distant metastases to other sites had little impact on survival time, studying the possible mechanisms and interventions for pleural effusion metastasis might provide possible directions for treatment. The patient reported in this paper was treated with multiple methods, including anti-angiogenic drugs, which might have played a role in delaying disease progression. It should be noted that the treatment combined with multiple methods would be a promising direction for future research.

Some of the limitations of this study were the small sample size and the retrospective design. Given the rarity of pulmonary NMC, the 62 cases reported in this study were sourced from 29 articles spanning a 13-year period. Despite our meticulous efforts to extract information comprehensively and reflect patient baseline data exhaustively, it was imperative to acknowledge the inherent potential for heterogeneity, particularly in the realms of treatment modalities and diagnostic criteria. Therefore, strengthening animal experiments and clinical trials might provide direction for future treatments.

## 6 Conclusion

While NMC could be diagnosed through positive NUT immunohistochemical staining or FISH testing for NUT gene mutations, the disease was typically diagnosed at an advanced stage, leading to reduced survival. Therefore, increasing awareness of this disease might help clinicians and pathologists to make an early diagnosis, potentially extending patients’ survival. As genetic mutations play a key role in the pathophysiology of the disease, intensifying efforts in the foundational and clinical evaluation of targeted therapies -exemplified by BET inhibitors- and a range of combinatorial treatments, would offer substantial potential in furnishing a robust theoretical foundation. This, in turn, could significantly contribute to the development of standardized therapeutic protocols. The current study highlights the negative impact of male gender and pleural effusion on the prognosis of pulmonary NMC. Additional investigation of these factors might provide new insights for disease management and treatment.

## Data availability statement

The original contributions presented in the study are included in the article/**Supplementary Material**. Further inquiries can be directed to the corresponding author.

## Author contributions

DZ: Data curation, Formal analysis, Funding acquisition, Methodology, Software, Writing – original draft. WC: Conceptualization, Formal analysis, Investigation, Methodology, Writing – review & editing. SZ: Conceptualization, Investigation, Supervision, Writing – review & editing. YW: Data curation, Investigation, Methodology, Software, Writing – review & editing. ZP: Data curation, Investigation, Methodology, Supervision, Writing – review & editing. JZ: Data curation, Formal analysis, Investigation, Supervision, Visualization, Writing – review & editing. KH: Formal analysis, Funding acquisition, Methodology, Resources, Writing – review & editing.
