# Supplementary material for: Primary pulmonary nuclear protein of the testis midline carcinoma: case report and systematic review with pooled analysis
Source: Front Oncol. 2024 Jan 9;13:1308432. doi: 10.3389/fonc.2023.1308432 (PMC10803636; doi:10.3389/fonc.2023.1308432)
Supplement: Supplementary file 1 [file Table_1.docx]

**Suppl 1. Clinicopathological Features and Outcome of the 62 Cases**

| Author, year | case | Gender/Age | Smoking | Primary location | Tumor size | Pleural effusion | Clinical presentation | Original Diagnosis | Treatment involved | Overall survival& (m) |
| --- | --- | --- | --- | --- | --- | --- | --- | --- | --- | --- |
| Ours | 1 | F/23 | N | NM | NM | Y | Cough, Shortness of breath, Chest pain | SCC | CRT+TT+IT | 7 |
| Zhang, 2021[8] | 2 | M/16 | NM | RUL | 6.3 | N | No symptom | NM | CT | 3 |
| Taniyama, 2014[9] | 3 | M/24 | NM | NM | NM | NM | NM | NSCLC | CT | 7.2 |
|  | 4 | F/29 | NM | NM | NM | NM | NM | NSCLC | CT | 3.6 |
| Lemelle, 2017[10] | 5 | F/15 | NM | NM | 5-10 | NM | NM | PNT | CRT | 17 |
|  | 6 | F/16 | NM | NM | 5-10 | Y | NM | SCC | CRT+TT | 9 |
|  | 7 | M/13 | NM | NM | NM | Y | NM | ES | CT | 4 |
|  | 8 | M/14 | NM | NM | > 10 | Y | NM | UC | CT | 2 |
|  | 9 | F/28 | NM | H | > 10 | Y | NM | NMC | CT | 1 |
|  | 10 | F/19 | NM | NM | > 10 | NM | NM | NMC | CT | 4 |
|  | 11 | M/35 | NM | NM | 5-10 | NM | NM | NMC | TT | 4 |
|  | 12 | F/16 | NM | NM | NM | NM | NM | UC | CT | 3 |
| Karakuş, 2017[11] | 13 | F/6 | NM | NM | NM | NM | Dyspnea. Cough, Weight loss | NM | CT | 6 |
| Al, 2017[12] | 14 | M/27 | N | LUL | NM | N | Cough | PNT | CRT | 5 |
| Tanaka, 2012[13] | 15 | M/14 | N | LLL | 7 | Y | Cough, Chest pain | UC | CRT+ST | 12 |
|  | 16 | F/7 | NM | LLL | NM | N | Cough, back pain | UC | CRT | 4 |
| Ueki, 2014[14] | 17 | F/12 | NM | RLL | 5 | N | back pain, Weight loss | NM | NM | 22 |
| Nelson,2010[15] | 18 | M/10 | NM | LLL | 10 | N | Chest pain | EC | ST+CT | 10 |
| Xie, 2020[16] | 19 | M/23 | Y | RUL | 5.5 | Y | NM | EC | ST | 1.5 |
|  | 20 | M/53 | Y | RUL | 5.4 | Y | NM | NMC | CT | 4.1 |
|  | 21 | F/30 | N | RLL | 4.7 | Y | NM | SCC | CT | 3 |
|  | 22 | M/25 | Y | RLL | 12.7 | Y | NM | NMC | CT | 1.5 |
|  | 23 | M/74 | Y | H | 5.3 | N | NM | NMC | RT | 19.5 |
|  | 24 | F/58 | N | H | 4 | N | NM | NMC | CT | 26.7 |
|  | 25 | F/31 | N | H | 3 | N | NM | NMC | CT | 12 |
| Stathis, 2016[17] | 26 | F/39 | NM | NM | 3 | N | NM | UC | ST+RT | 19 |
|  | 27 | M/66 | NM | RUL | 8 | NM | NM | SCLC | CRT | 18 |
| Virarkar, 2021[18] | 28 | F/17 | NM | LLL | NM | Y | back pain | NM | CRT | 6 |
|  | 29 | F/51 | NM | RUL | 2.8 | Y | Cough | NM | CRT+TT | 6 |
|  | 30 | M/19 | NM | RLL | 10 | Y | Weight loss | NM | CRT+TT | 3 |
|  | 31 | M/23 | NM | RUL | 7 | N | Weight loss | NM | RT+TT | 5 |
|  | 32 | F/42 | NM | LUL | 6 | Y | Dyspnea | NM | CRT | 20 |
|  | 33 | F/17 | NM | RLL | 10 | Y | back pain | NM | CT | 2 |
|  | 34 | F/27 | NM | LLL | 8 | Y | Cough | NM | CT+TT | 10 |
|  | 35 | M/33 | NM | LUL | 3 | Y | back pain | NM | CRT+TT | 1 |
|  | 36 | F/32 | NM | H | 3 | N | NM | NM | CRT | 12 |
|  | 37 | M/51 | NM | NM | 7 | Y | Shortness of breath | NM | CRT+TT | 2 |
|  | 38 | F/47 | NM | NM | NM | Y | Cough | NM | CRT+TT | 5 |
| Zhou, 2020[19] | 39 | M/26 | NM | RLL | 2 | NM | Shortness of breath, Cough | SCLC | CT | 5 |
|  | 40 | M/69 | NM | H | 5 | NM | Shortness of breath | SCLC | CT | 6 |
| Lee, 2017[20] | 41 | M/34 | Y | NM | NM | Y | NM | NM | CT | 4 |
|  | 42 | F/33 | N | NM | NM | Y | NM | NM | CT | 8 |
| Policarpio, 2015[21] | 43 | M/32 | N | RLL | 9 | Y | Dyspnea | NM | Palliative care | 1 |
| Kuroda, 2015[22] | 44 | F/36 | NM | NM | 7.5 | NM | Cough | NM | CT | 10 |
| Shenoy, 2019[23] | 45 | M/25 | Y | NM | NM | Y | Dyspnea | NM | Palliative care | 0.5 |
|  | 46 | M/29 | NM | LUL | 7.0 | Y | Cough, Dyspnea | NM | CT | 2 |
| Engleson, 2006[24] | 47 | F/30 | NM | H | NM | Y | back pain, Weight loss | NM | CRT | 3.5 |
| Suzuki, 2015[25] | 48 | F/36 | NM | LLL | NM | N | Cough, Shortness of breath | NM | CRT | 9 |
| Shatavi, 2016[26] | 49 | F/23 | NM | NM | NM | N | Dyspnea | NM | CT | 21 |
| Puliyel, 2014[27] | 50 | F/13 | NM | RLL | 8.0 | Y | Cough, Dyspnea | NMC | CT | 6 |
| Raza, 2015[28] | 51 | M/36 | Y | H | 9 | Y | Dyspnea | NM | CRT | 10 |
| Zhao, 2022[29] | 52 | M/40 | NM | RUL | 11 | N | Chest pain | NM | CRT+TT+IT | 4.7 |
| Jiang, 2022[30] | 53 | M/33 | Y | LLL | 6 | Y | Cough, Chest pain | NM | CRT+TT | 4 |
| Cao, 2017[31] | 54 | M/48 | Y | H | 8 | N | Shortness of breath | SCC | ST+RT | 6 |
| Teo, 2010[32] | 55 | F/22 | Y | RUL | 13 | Y | Cough, Chest pain | NM | CT | 3 |
| Ma, 2018[33] | 56 | M/10 | NM | H | 10 | N | Cough | NM | NM | 1 |
| Chang, 2021[34] | 57 | F/41 | N | RUL | 3.3 | NM | Cough | NM | CT | 3 |
|  | 58 | M/49 | Y | RLL | 5.5 | NM | Chest pain | NM | CT | 3 |
|  | 59 | M/34 | Y | RML | 2.3 | NM | Chest pain | NM | CRT | 12 |
|  | 60 | M/32 | Y | LLL | 6.5 | NM | Cough | NM | CT | 3 |
| Zhang, 2022[35] | 61 | M/41 | N | RML | 3.9 | NM | NM | NM | RT | 1 |
| Parikh, 2013[36] | 62 | M/36 | N | H | 6 | NM | Cough, Chest pain | NM | CT+TT | 1 |

M, male; F, female; NM, not mentioned; SCC, squamous cell carcinoma; SCLC, small cell lung carcinoma; NSCLC, nonsmall cell lung carcinoma; PNT, Primary neuroectodermal tumor; ES, Ewing’s sarcoma; UC, Undifferentiated carcinoma; NMC, NUT midlin carcinoma; EC, Epithelial carcinoma; RUL, right upper lobe; RML, right middle lobe; RLL, right lower lobe; H, hilum; LUL, left upper lobe; LLL, left lower lobe; ST, surgical treatment; CRT, chemoradiotherapy; CT, chemotherapy; RT, radiotherapy; TT, targeted therapy.
